# Seasonal Characterization of the Endophytic Fungal Microbiome of Mulberry (*Morus* spp.) Cultivars Resistant and Susceptible to Sclerotiniosis

**DOI:** 10.3390/microorganisms9102052

**Published:** 2021-09-28

**Authors:** Weifang Xu, Fei Wang, Ruolin Wang, Yuan Sui, Zeyang Zhou, Jie Xie, Zhonghuai Xiang

**Affiliations:** 1 State Key Laboratory of Silkworm Genome Biology, Key Laboratory of Sericultural Biology and Genetic Breeding in Ministry of Agriculture, College of Sericulture, Textile and Biomass Sciences, Southwest University, Chongqing 400715, China; xwf0108wl@sina.com (W.X.); w1994f0806@sina.com (F.W.); xiaoruo0348@sina.com (R.W.); zyzhou@swu.edu.cn (Z.Z.); xbxzh@swu.edu.cn (Z.X.); 2 Anhui Province Key Laboratory of Research & Development of Chinese Medicine, School of Pharmacy, Anhui University of Chinese Medicine, Hefei 230012, China; 3 Chongqing Key Laboratory of Economic Plant Biotechnology, College of Landscape Architecture and Life Science/Institute of Special Plants, Chongqing University of Arts and Sciences, Chongqing 402160, China; suiyuan-mine@163.com; 4 College of Life Science, Chongqing Normal University, Chongqing 400047, China

**Keywords:** endophytic fungi, community composition, seasonal shift, mulberry cultivars, resistance to sclerotiniosis

## Abstract

The endophytic microbiome is thought to play an important role in promoting plant growth and health. Using culture-independent and culture-dependent protocols, this study characterized the seasonal shifts in the endophytic fungal microbiota of four mulberry (*Morus* L.) cultivars having different levels of resistance to mulberry fruit sclerotiniosis. Core endophytes can be obtained by two approaches, and they were divided into two clusters by season. Spring samples harbored higher operational taxonomic units (OTUs) and α-diversity, while autumn samples had more sequences or isolates of the fungal class *Dothideomycetes* with the representative orders *Capnodiales* and *Pleosporales*. While comparing different mulberry cultivars, we found that the total number of OTUs in susceptible cultivars was higher than that of resistant cultivars, and *Cladosporium* sp. were observed in all. Notably, the causal agent of fruit sclerotiniosis (*Scleromitrula shiraiana*) was only detected in susceptible cultivars. Collectively, our work elucidated significant variations in the mulberry endophytic microbiome, mainly because of seasonal shifts, and the fact that the host cultivars and mulberry endophytic fungal community appeared to have a certain connection with the resistance level of mulberry fruit to sclerotiniosis. These results provided valuable information on the isolation and culturing of mulberry endophytes that could be applied to improve mulberry fruit production and health.

## 1. Introduction

Sericulture plays a key role in the Ancient Chinese Silk Road, and may also be an important component in the Belt and Road Initiative efforts today. Mulberry (*Morus* L.) is used as a unique food source for silkworms (*Bombyx mori*), as well as for ecological stabilization and as a medicinal substance [1]. Mulberry fruit is considered to be beneficial for human health as it is rich in amino acids, vitamins and minerals [2,3]. The use of mulberry fruit is expected to increase to address the needs of the sericulture industry. Mulberry fruit sclerotiniosis, however, seriously affects the quality and yield of fruit, resulting in economic loss. This disease is primarily caused by soil-borne fungal pathogens: *Ciboria shiraiana* [4], *Ciboria carunculoides* [5], *Scleromitrula shiraiana* [6], and *Sclerotinia sclerotiorum* [7]. These pathogens generally infect mulberry flowers in the spring (late February to early April in Chongqing, China) when mulberry trees bloom and the climate is suitable, and symptoms start in late April. It is noteworthy that, although the flowers remain infected, the symptoms of the disease are not expressed on the flowers, but only on the fruit. Exploiting efficient and environmentally friendly methods for the biological control of sclerotiniosis is essential for the sustainable development of the mulberry fruit industry and for the genetic improvement of mulberry fruit cultivars.

Endophytes are microorganisms that reside within plant tissues without any apparent adverse effect on the host plants [8]. They generally form a range of different relationships with their host, such as symbioses including mutualism and commensalism [9,10]. Studies of mulberry endophytes have been initially focused on biodiversity [11,12] and for antibiotic production [13,14,15]. Microbial communities inhabiting mulberry appear to affect plant growth and health positively [16,17]. Therefore, clarification of endophyte diversity in the mulberry can contribute to elucidating their function and potential role in developing sustainable production and management systems for the mulberry industry [18,19]. Many fungi living as endophytes or pathogens inside a host plant are associated with immune responses like the secretion of effectors, enzymes, and secondary metabolites [20]. Significant differences have been reported in the composition of the soil fungal community of different mulberry cultivars that are associated with differences in resistance to sclerotiniosis [21]. Research on the endophytic bacterial communities of four mulberry cultivars indicated that the correlation of the endophytic communities and the disease incidence of sclerotiniosis in susceptible mulberry cultivars was higher than in resistant cultivars [11]. Huang et al. found that the resistance of different mulberry varieties to mulberry fruit sclerotiniosis was quite different: “Chuan Sang No.7637” (*Morus alba* L.) has the strongest resistance to sclerotinia with a disease incidence of almost 0. “Xin Lunjiao” (*Morus atropurpurea* Roxb.) and “Hong Guo No.2” (*Morus atropurpurea* Roxb.), were two susceptible cultivars having a disease incidence of 46.18 and 56.8%, respectively [22]. In addition, we found that “Changguo Sang” (*Morus laevigata* Wall), with a disease incidence of 1.8%, was resistant to mulberry fruit sclerotiniosis based on the investigation of mulberry diseases in the past few years. Little knowledge is available, however, pertaining to the distribution of endophytic fungi in different mulberry cultivars and their potential role in resistance to sclerotiniosis.

Plant–microbe interactions depend on biotic and abiotic factors. Wagner et al. [23] reported that a combination of environmental factors and the genotype of the host plant *Boechera stricta* shapes the leaf and root microbiome of this wild perennial. The host genotype may affect the composition and function of endophytes, which in turn may affect plant response, pathogen colonization and behavior, and microbial genetic variation [24]. Endophytic composition of two transgenic maize genotypes differed in near-isogenic and non-transgenic maize genotypes [25]. Resistance breeding in the common bean has been reported to influence rhizosphere microbiome composition and function [26]. In plants, microbial communities have also changed across seasons, likely due to the seasonality of photosynthesis, temperature, humidity, and CO_2_ levels, which consequently affect host health [27,28]. For example, endophytic fungal communities in the Hawaiian tree, *Metrosideros polymorpha*, are affected by environmental factors such as precipitation and temperature [29]. Little information exists, however, on the composition of endophytic fungi among different mulberry cultivars.

Endophytes have been typically isolated and analyzed using traditional culture-dependent approaches in the past few decades. In recent years, the advent of molecular tools has provided new insights into environmental microbiome investigations and has resulted in the identification of a number of uncharacterized endophytic microorganisms [30,31,32,33]. Next-generation sequencing (NGS) technologies have provided a high resolution approach to the comprehensive assessment of plant endophytic communities and studies of endophytic diversity [34]. Culture-independent methods can detect unculturable endophytic colonizers of plants, as well as those endophytes that grow so slowly that they are missed by traditional culture methods. Few studies have been conducted on the endophytic fungi in mulberry trees by culture-independent methods such as NGS.

Therefore, this study analyzed endophytic fungal communities of four mulberry cultivars with different resistance to sclerotiniosis in spring and autumn. The two resistant cultivars were “Chuan Sang No.7637” and “Changguo Sang”, and the two susceptible cultivars were “Hong Guo No.2” and “Xin Lunjiao” [22]. Seasonal shifts in the composition of the endophytic fungal community were analyzed by both culture-independent and culture-dependent methods. The objective was to better understand the factors that might affect the assemblage of the mulberry endophytic microbiome and to further establish a solid foundation for exploring the biological control of mulberry fruit sclerotiniosis.

## 2. Materials and Methods

### 2.1. Sample Collection and Processing

Healthy branches from four healthy mulberry cultivars, “Changguo Sang” (CG) and “Chuan Sang No.7637” (CQ), which are resistant to mulberry fruit sclerotiniosis, and “Hong Guo No.2” (HG) and “Xin Lunjiao” (XL), which are susceptible to mulberry fruit sclerotiniosis, were collected. Among the four, CG was sampled from a planting at Southwest University in Chongqing, China (29°49′1″ N, 106°24′57″ E), and the other three cultivars were sampled from a planting located at Sericulture Science and Technology Research Institute, Chongqing, China (29°50′39″ N, 106°25′55″ E). The two sampling sites share the same climatic conditions near the Jialing River. The sampling method was based on procedures described by Ou et al. [11]. Branch samples from the four cultivars were from two-year-old mulberry trees and were collected in the spring (April 2016) and autumn (September 2016), respectively. The branches were approximately 50.0 cm in length and 1.5–2.0 cm in diameter. Eight branch samples (2 seasons × 4 cultivars) were placed in sterile polythene bags, immediately transported back to the laboratory and stored at 4 °C until further processing.

Plant materials were washed with tap water, dried naturally, and then cut into approximately 4.0 cm segments [35,36], which were then immersed in 75% ethanol and flamed once for surface disinfection. The efficiency of the disinfection was evaluated by rolling the treated branches segments on potato dextrose agar (PDA) and incubating them at 22 °C for 1 week. Only those mulberry segments that did not result in microbial growth on the PDA plates after incubation were used for subsequent endophyte enrichment and isolation.

### 2.2. Endophyte Enrichment

Ten surface-sterilized segments in each cultivar were randomly selected, pooled, and served as one replicate for endophyte enrichment. All eight samples were processed in three biological replicates. The process of endophyte enrichment was conducted as previously described [37,38]. Approximately 5 g of surface-sterilized tissue was chopped and homogenized in a sterilized plant tissue homogenizer using sterilized distilled water. The homogenate was then filtered through two layers of gauze and centrifuged at 200× *g* for 5 min at 4 °C. Next, the supernatant was collected into a new sterile tube, and NaCl and 10% sodium dodecyl sulfate (SDS) were added to a final concentration of 0.9% and 0.063% (*w*/*v*), respectively. The mixture was shaken slightly and then left to incubate for 1 h. After settling, the upper phase was transferred to a new tube and centrifuged for 10 min at 4 °C at 5000× *g*. The supernatant was then removed and 200 mL of sterilized distilled water was added in preparation to harvest a pellet. The NaCl and SDS were added to the suspension, which was centrifuged again as described above. The procedure was repeated until at least 100 mg of precipitate was obtained and resuspended in 1 mL of TE buffer (1 mM EDTA, pH 8.0; 10 mM Tris-HCl, pH 8.0) at 4 °C.

### 2.3. DNA Extraction and ITS Gene Sequencing

DNA was extracted using a protocol described by Murray et al. [39,40,41]. Total genomic DNA was extracted from the sampled pellet using the modified cetyltrimethylammonium bromide (CTAB) method. Lysozyme and RNaseA were added to the suspension enriched in microorganisms and the mixtures were incubated at 37 °C for 10 min. The mixtures were then treated with 6 μL Proteinase K and 60 μL 10% SDS and incubated at 55 °C for 20 min until the liquid became clear. Subsequently, 200 μL 5 M NaCl was added and the solution was gently mixed. An equal volume of CTAB extraction buffer (2% (*w*/*v*) cetyltrimethylammonium bromide, CTAB; 100 mM Tris-HCl, 1.4 M NaCl, 20 mM EDTA, 1.5% polyvinyl-pyrrolidone, PVP; 0.5% 2-mercaptoethanol) was added and mixed by inversion, followed by incubation in a 65 °C water bath for 20–45 min. Next, an equal volume of cold phenol/chloroform/isoamyl alcohol solution (25:24:1; *v*/*v*/*v*) was added and the solution was gently mixed again and centrifuged at 16,000× *g* for 15 min at 4 °C. The resulting supernatant was collected into new tubes and an equal volume of chloroform/isoamyl alcohol solution (24:1; *v*/*v*) was added to each tube. The upper phase was then collected and transferred to a new tube. After centrifugation at 16,000× *g* for 15 min at 4 °C, 3 M NaAc representing 10% of the total volume and 2.5-fold pre-cooled ethanol were added. The samples were then stored at −20 °C for 12 h and subsequently centrifuged (16,000× *g*, 15 min). The supernatant was discarded and the DNA pellet was washed with 70% ethanol, centrifuged at 16,000× *g* for 5 min at 4 °C, and then air-dried. Lastly, the DNA was resuspended in 30 μL of TE buffer and stored at −20 °C. The final DNA concentration and purity of each sample were monitored on 1% agarose gels.

The fungal primers ITS1 (5′-CTTGGTCATTTAGAGGAAGTAA−3′) and ITS2 (5′-GCTGCGTTCTTCATCGATGC-3′) targeting the ITS1 regions of fungal rRNA genes were used to generate amplicons for sequencing [42]. Replicate PCR products of the same sample were assembled within a PCR tube. PCR reactions were performed in a 20 μL mixture containing 4 μL of 5 × FastPfu Buffer, 2 μL of 2.5 mM dNTPs, 0.8 μL of each primer (5 μM), 0.4 μL of FastPfu Polymerase, 0.2 μL of BSA and 10 ng of template DNA. The PCR reactions were conducted using the following program: 95 °C for 3 min, 95 °C 30 s, 55 °C 30 s and 72 °C 45 s for 35 cycles, and a final extension of 72 °C for 10 min. Products were purified and recovered by agarose gel electrophoresis. The recovered products were then quantified with Pico Green using a QuantiFluor™-ST (Promega, Madison, WI, USA), and equimolar concentrations of PCR products for each sample were pooled. The PCR products were extracted from a 2% agarose gel and purified with an AxyPrep DNA Gel Extraction Kit (Axygen Biosciences, Union City, CA, USA). Prior to MiSeq sequencing, the concentration and quality of the purified PCR product was checked using a QuantiFluor™-ST (Promega, Madison, Wisconsin, USA) according to the manufacturer’s protocol. Purified amplicons were pooled in equimolar and paired-end sequenced (2 × 300) on an Illumina MiSeq platform (Illumina, San Diego, CA, USA) according to standard protocols described by Majorbio Bio-Pharm Technology Co. Ltd. (Shanghai, China). The raw reads were deposited into the NCBI Sequence Read Archive (SRA) database (Accession Number: SRP165744).

### 2.4. Culturing of Endophytic Fungi

The method used to culture endophytic fungi was based on procedures described by Xie et al. [43]. Surface sterilized mulberry stem segments were placed into sterile Petri dishes, aseptically sectioned into 3 sections of smaller pieces, and distributed onto the surface of PDA, Gauze No.1 agar (GA), and Water agar (WA). The plates were incubated at 22 °C for 4 weeks and observed daily. After hyphae developed from the edge of a stem piece, the tips were picked and transferred to the new plates. The purified isolates were stored in a 50% glycerol solution at −80 °C. Genomic DNA was extracted from fungal isolates by using Prep Man Ultra Sample Preparation Reagent (Applied Biosystems, Palo Alto, CA, USA). The primers of ITS1 (5′-TCCGTAGGTGAACCTGCGG-3′) and ITS4 (5′-TCCTCCGCTTATTGATATGC-3′) were used to amplify the internal transcribed spacer (ITS). PCR reaction conditions were used as follows: initial denaturation at 94 °C for 4 min, followed by 30 cycles of denaturation at 94 °C for 30 s, annealing at 55 °C for 45 s, and elongation at 72 °C for 1 min; and final extension at 72 °C for 8 min. The PCR products were sequenced in Sangon Biotech Co, Ltd. (Shanghai, China). All the cultured isolates were identified at the genus level in the NCBI GenBank database according to the BLAST results of ITS. In addition, the taxonomic database of NCBI was used to classify all the endophytic fungal strains, including the level of phylum, class, order, family, genus and species. Sequence data from this study can be found in the NCBI data libraries under accession numbers MH884069-MH884177.

### 2.5. Bioinformatics and Statistical Analysis

To facilitate comparing the differences in fungal community structure in different mulberry samples, the eight groups were established considering the influence of both cultivars and seasons on the fungal community structure: (1) SH–HG in spring; (2) SX–XL in spring; (3) SQ–CQ in spring; (4) SC–CG in spring; (5) AH–HG in autumn; (6) AX–XL in autumn; (7) AQ–CQ in autumn; and (8) AC–CG in autumn. In addition, 4 groups were also built that only took into account the cultivar factor. These groups were based on combining spring and autumn samples together of each cultivar as described above (XL, HG, CQ, and CG).

DNA samples from these groups were sequenced on the Illumina platform the Majorbio-Shanghai. Raw fastq files were demultiplexed, quality-filtered by trimmomatic and merged by FLASH (version 1.2.11) using the following criteria: (i) Reads that were truncated at any site received an average quality score <20 over a 50 bp sliding window. (ii) Exactly matched primers that allowed 2-nucleotide mismatches and reads containing ambiguous bases were removed. (iii) Sequences with an overlap longer than 10 bp were merged based on their overlap sequence. Operational taxonomic units (OTUs) were clustered using a 97% similarity cutoff with UPARSE (version 7.0.1090) and chimeric sequences were identified and removed using the UCHIME algorithm (Available online: http://drive5.com/usearch/manual/dereplication.html (accessed on 27 August 2019)). The taxonomy of each ITS sequence was determined using the RDP Classifier algorithm (version 2.11, Available online: https://sourceforge.net/projects/rdp-classifier/ (accessed on 8 September 2019)) against the UNITE (Release 7.0, Available online: http://unite.ut.ee/index.php (accessed on 8 September 2019)) ITS database using a confidence threshold of 70%. The raw data of metagenomic sequencing were processed and analyzed on the free online platform of Majorbio I-Sanger Cloud Platform (Available online: www.i-sanger.com (accessed on 8 September 2019)). Based on the sobs index on the OTU level, rarefaction curves were performed on each sample to assess the adequacy of the sampling. Beta diversity analysis, principal coordinate analysis (PCoA) and hierarchical clustering based on the distance matrix with calculation of the Bray–Curtis algorithm were used to analyze differences. Alpha diversity was calculated including the Sobs, Chao, Shannon, and Simpson indices, using Mothur software (version 1.30.1), to compare the diversity and richness of the endophytic community in different samples. Among these indices, the former two are typically used to evaluate community richness, while community diversity can be calculated by the Shannon and Simpson index. Community bar plots and Venn diagrams were used to illustrate the variation and richness in community structure. The mean of the top 10 for richness, evenness and diversity was applied for Welch’s *t*-test to determine significant differences among the samples [34,44,45].

## 3. Results

### 3.1. Biodiversity of Endophytic Fungi Based on ITS Amplicon Sequencing

**Figure 1:**
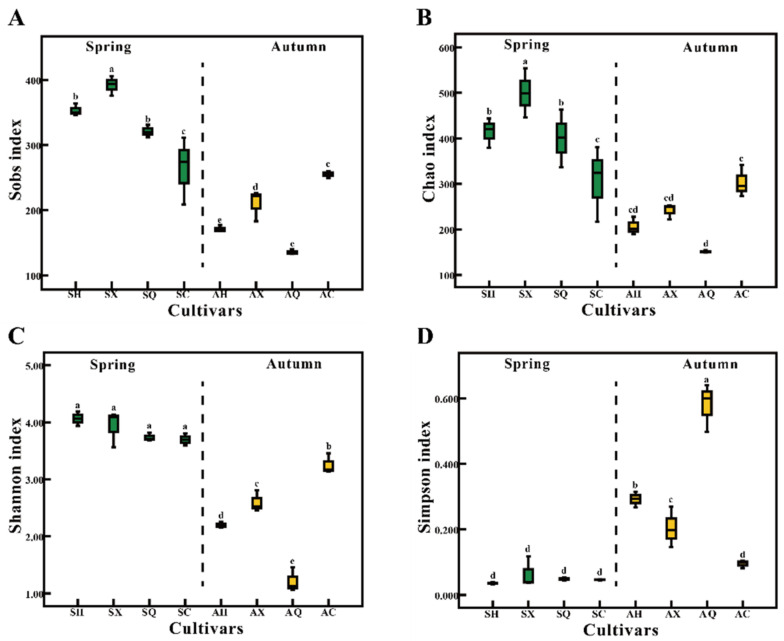
Alpha diversity analysis of the endophytic fungal communities of four different mulberry cultivars in spring and autumn. (**A**) Sobs; (**B**) Chao; (**C**) Shannon; (**D**) Simpson. The *x*-axis indicates the mulberry cultivar and the *y*-axis represents the observed value of different indices based on OTU abundance. *n* = 3 for each cultivar. Bars with the different letters indicate a significant difference between means by one-way ANOVA and Duncan’s multiple test (*p* < 0.05). Values represent the mean. Error bars indicate ± standard deviation. Abbreviations: SH, SX, SQ, and SC represent fungal communities from “Hong Guo No.2”, “Xin Lunjiao”, “Chuan Sang No.7637”, and “Changguo Sang” in spring, respectively. AH, AX, AQ, and AC represent fungal communities from “Hong Guo No.2”, “Xin Lunjiao”, “Chuan Sang No.7637” and “Changguo Sang”, respectively, in autumn.

After quality control and filtering, Illumina MiSeq sequencing analysis produced 803,114 sequences of good quality. The total number of detected OTUs at 97% sequence similarity was up to 1038 in the 24 DNA samples (2 seasons × 4 cultivars × 3 replicates). The rarefaction curves tended to approach the saturation plateau in all four mulberry cultivars, indicating that the sequencing depth was adequate (Figure S1). Simultaneously, the Sobs, Chao, Shannon, and Simpson indices calculated from the fungal OTUs of four mulberry cultivars in two seasons indicated that a greater alpha diversity of fungal OTUs were detected in the spring than in the autumn samples regardless of cultivar (Figure 1). For the richness indices, the average value of both Sobs and Chao in spring samples exhibited a clear upward trend compared to the autumn samples (Sobs: SH (353.33) > AH (171.33), SX (392.00) > AX (210.33), SQ (321.00) > AQ (135.67), SC (264.67) > AC (255.00); Chao: SH (414.15) > AH (206.40), SX (499.73) > AX (241.71), SQ (400.69) > AQ (151.42), SC (307.17) > AC (303.44)) (Figure 1 and Table S1). This trend also appeared on the Shannon diversity index (Figure 1 and Table S1). In regards to cultivars, the susceptible cultivars HG (Chao, 414.15; Shannon, 4.06) and XL (Chao, 499.73; Shannon, 3.93) in spring samples exhibited higher richness and diversity than the resistance cultivars CQ (Chao, 400.69; Shannon, 3.74) and CG (Chao, 307.17; Shannon, 3.70) (Figure 1 and Table S1). This phenomenon, however, was not obvious in the autumn samples. These results indicated that season had great impact on community diversity, followed by the host cultivars.

### 3.2. Taxonomic Composition of Endophytic Fungi

**Figure 2:**
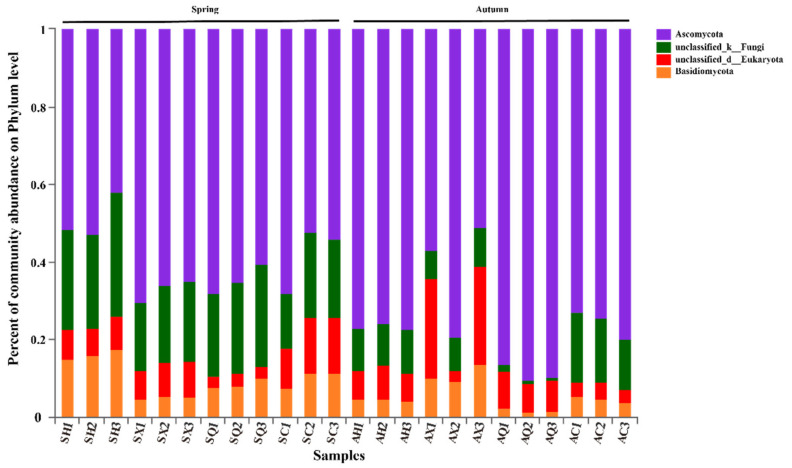
Relative abundance of the phyla of endophytic fungi in four mulberry cultivars in spring and autumn. The *x*-axis indicates communities of different samples and the *y*-axis represents the relative abundance within the total community. Abbreviations: SH, SX, SQ, and SC represent fungal communities from “Hong Guo No.2”, “Xin Lunjiao”, “Chuan Sang No.7637”, and “Changguo Sang” in spring, respectively. AH, AX, AQ and AC represent fungal communities from “Hong Guo No.2”, “Xin Lunjiao”, “Chuan Sang No.7637”, and “Changguo Sang”, respectively, in autumn. Numbers 1 to 3 refer to the replicates of each sample.

Based on high-throughput amplicon sequencing, members of the *Ascomycota* were the dominant fungal phylum across all samples, accounting for 67.78% of the total number of detected sequences. This was followed by *Basidiomycota* (7.68%), while the remaining OTUs could not be classified (Figure 2). Within the *Ascomycota*, the OTUs were largely identified as members of the classes *Dothideomycetes* (58.65%) and *Sordariomycetes* (2.63%). The *Dothideomycetes* were principally represented by members of the orders *Capnodiales* (30.89%) and *Pleosporales* (13.37%). *Sordariomycetes* was represented only by the order *Hypocreales* (2.47%). Moreover, members of the order *Capnodiales* were mainly comprised of *Cladosporium* sp. (25.38%).

Based on results from the culture-dependent method, a total of 109 isolates were obtained from all of the samples, comprising 1 phylum, 3 classes, 10 orders, 13 families, and 19 genera. On the phylum level, all isolates belonged to *Ascomycota*. The total number of isolates grouped into three classes, *Dothideomycetes* (55), *Sordariomycetes* (50), and *Eurotiomycetes* (4). Classification of the dominant orders included: *Hypocreales* (30), *Pleosporales* (28), and *Capnodiales* (24) (Table S2). *Cladosporium* sp. (24) and *Fusarium* sp. (29) were the primary genera. Overall, the core mulberry endophytic microbiome was easily isolated and cultured.

### 3.3. Differences in the Spring and Autumn Communities of Endophytic Fungi

**Figure 3:**
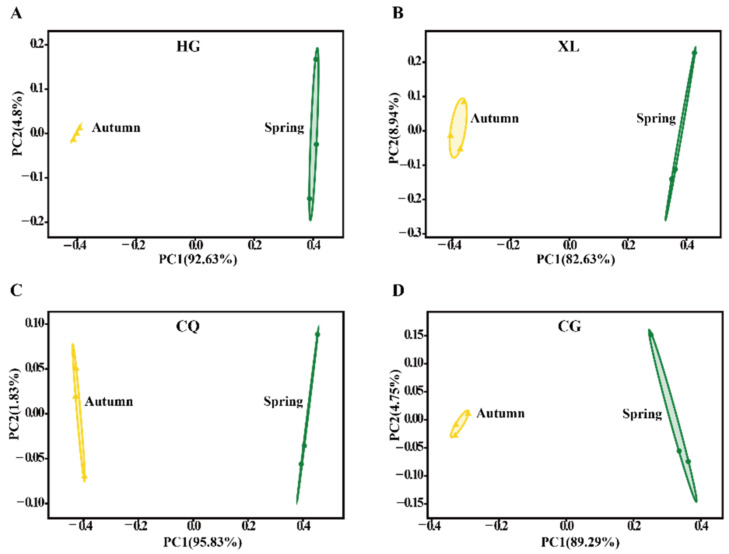
PCoA analysis based on Bray Curtis index of spring and autumn samples from each cultivar. HG (**A**), XL (**B**), CQ (**C**), and CG (**D**) represent fungal communities from “Hong Guo No.2”, “Xin Lunjiao”, “Chuan Sang No.7637”, and “Changguo Sang”, respectively. *n* = 3 for each sample. Spring samples are represented by green dots and autumn samples by yellow triangles.

**Figure 4:**
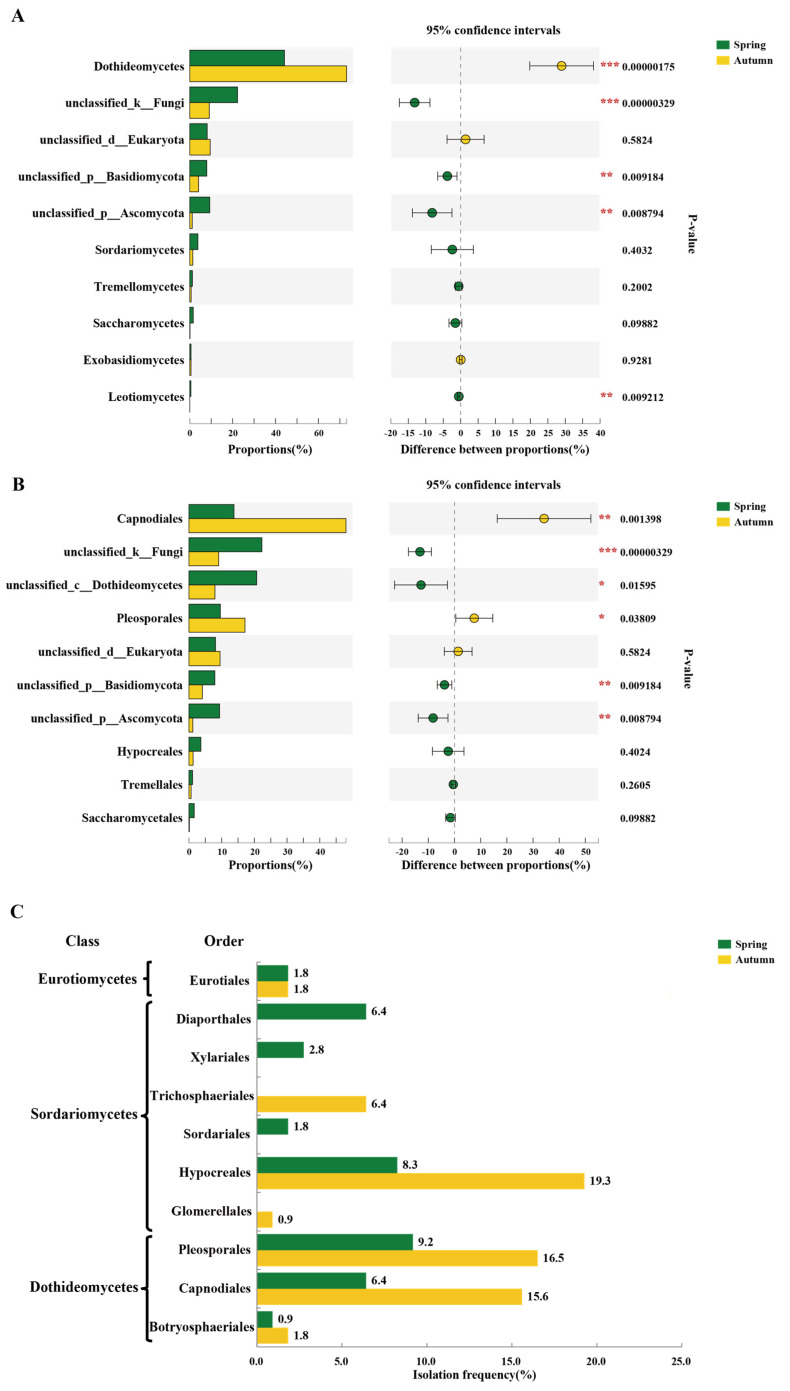
Comparison of the relative abundance of the most abundant taxa of endophytic fungi of mulberry in two seasons. (**A**) The 10 most abundant fungal classes in spring and autumn samples based on ITS amplicon sequencing. (**B**) The 10 most abundant fungal orders in spring and autumn samples based on ITS amplicon sequencing. (**C**) Taxonomic distribution (class-level and order-level) of isolates in spring and autumn samples based on the culture-dependent method. Different colors represent different groups. The dot indicates the percentage difference in abundance between the two groups, the color of the dot is presented as the group color with a large abundance. *** *p* < 0.001, ** *p* < 0.01, * *p* < 0.05. Welch’s *t*-test and *p*-value were corrected by the FDR method. The isolation frequency (IF) was calculated using the formula: IF (%) = the number of isolates of a certain taxa/the total number of isolates ×100%.

Both PCoA and hierarchical cluster analysis indicated that samples collected in the spring and autumn differed significantly in their fungal communities (Figure 3 and Figure S2). The number of observed fungal OTUs declined from 813 in spring to 652 in autumn. The number of unique OTUs in the combined samples also declined from 386 in the spring to 225 in the autumn (Figure S3A). Additionally, amplicon-based community composition analysis indicated that the most dominant fungal phylum detected in the spring samples was *Ascomycota* (59.61%), while it increased to 75.96% in the autumn samples. *Basidiomycota* represented 9.96 and 5.41% in the spring and autumn samples, respectively. At the class level, members of the *Dothideomycetes* showed a large increase from 44.17 in the spring samples to 73.13% in autumn samples. In contrast, *Sordariomycetes*, *Saccharomycetes,* and *Tremellomycetes* accounted for 3.82, 1.63, and 1.24% in the spring samples, respectively, but decreased to 1.43, 0.12, and 0.67% in the autumn samples, respectively (Figure 4A). Additionally, the predominant orders detected in the autumn were *Capnodiales* (48.00%) and *Pleosporales* (17.14%), which declined to 13.78 and 9.6% in the spring samples, respectively (Figure 4B). Overall, a comparison of the communities in the spring and autumn samples indicated that fungal composition and distribution were not equivalent in the different seasons.

Based on the culture-dependent data, members of the *Capnodiales* (15.6%) and *Pleosporales* (16.3%) were isolated at a higher frequency in the autumn samples than in the spring samples, indicating that they were readily cultured. The isolation frequency of members of the *Hypocreales* (19.3%) was the highest in autumn, when their relative abundance was only 1.28% (Figure 4C). These analyses also revealed distinct differences in spring and autumn endophytic fungal communities.

### 3.4. Comparative Analysis of the Composition of Endophytic Fungi among the Four Mulberry Cultivars

**Figure 5:**
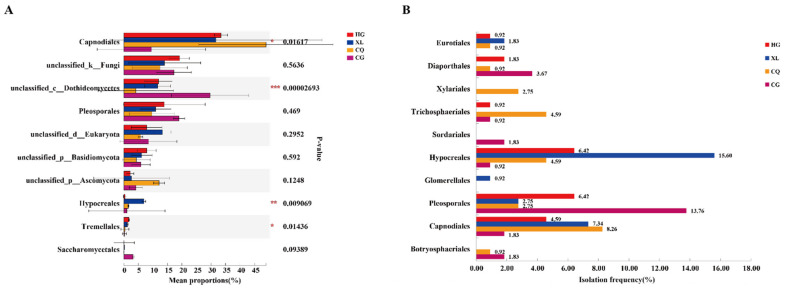
The relative abundance of the most abundant endophytic fungi in four mulberry cultivars. (**A**) The 10 most abundant fungal orders in four mulberry cultivars based on ITS amplicon sequencing. (**B**) Taxonomic distribution (order-level) of isolates from four mulberry cultivars samples based on the culture-dependent method. Different colors represent different groups. *** *p* < 0.001, ** *p* < 0.01, * *p* < 0.05. One-way ANOVA and *p*-value were corrected by the FDR method. The isolation frequency (IF) was calculated using the formula: IF (%) = the number of isolates of a certain taxa/the total number of isolates ×100%.

Regardless of the impact of the season, the total number of observed fungal OTUs was the highest in XL (710), followed by HG (594), CG (587), and CQ (563) (Figure S3B). The number of OTUs shared between susceptible cultivars (HG and XL, 458) was much higher than the number shared between resistant cultivars (CQ and CG, 355). Moreover, the number of unique OTUs in the susceptible cultivar XL (131) was higher than the number in HG (74), while the number of unique OTUs in the resistant cultivar CG (98) was higher than that in CQ (70) (Figure S3B). There were no obvious differences in the variability of the fungal community of the four mulberry cultivars at the phylum and class level. At the order level, however, the distribution of fungal communities in the four cultivars exhibited some differences. Notably, the resistant cultivar CQ had the highest relative abundance of *Capnodiales*, while CG had the lowest (CQ, 48.95 vs. CG, 9.48%). In the susceptible mulberry cultivars, a similar level of relative abundance was observed in members of the *Capnodiales* (HG, 33.44 vs. XL, 31.68%). In contrast, the resistant cultivar CG had the highest relative abundance of *Pleosporales*, while CQ had the lowest (CG, 18.99 vs. CQ, 9.48%). The relative abundance of *Pleosporales* in HG was similar to that in XL (HG, 13.89 vs. XL, 10.94%). Interestingly, *Saccharomycetales* was detected almost exclusively in CG (CG, 3.20%) (Figure 5A). These analyses based on the culture-independent data indicated that the relative abundance of the various taxa in susceptible cultivars was similar, while the relative abundance of taxa in resistant cultivars varied significantly. Moreover, members of the *Hypocreales, Pleosporales,* and *Capnodiales* were readily isolated by culture methods from XL (15.6%), CG (13.76%), and CQ (8.26%), which may be related to their higher abundance within these cultivars (Figure 5B).

### 3.5. Influence of Seasonal Shifts on the Fungal Composition of Mulberry Cultivars with Different Levels of Resistance to Sclerotiniosis

**Figure 6:**
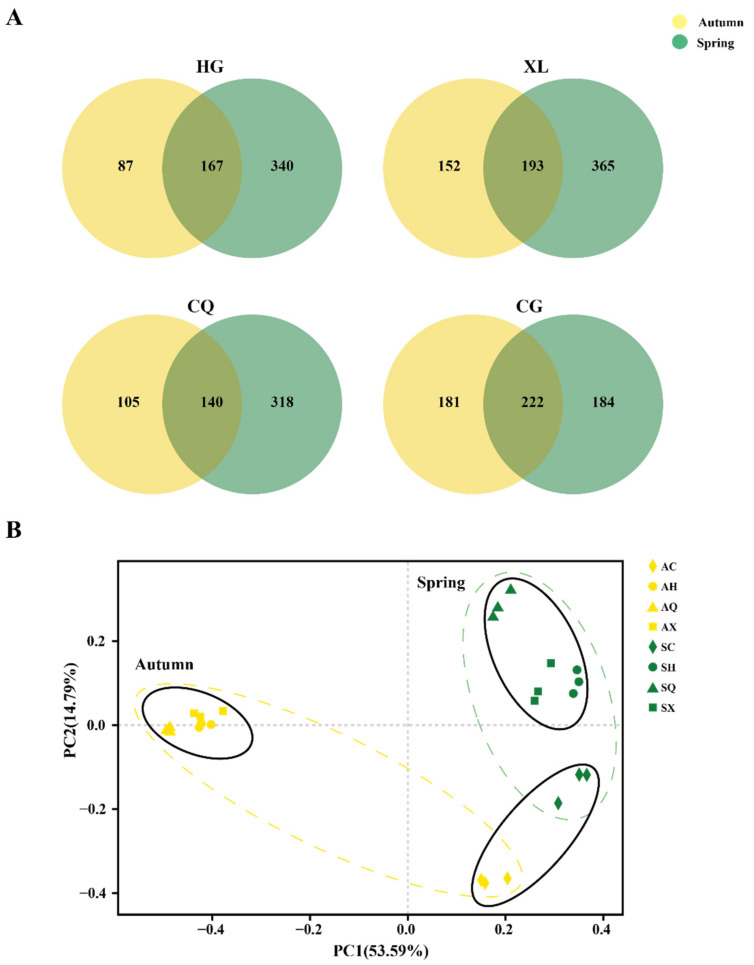
Comparisons of diversity in the spring and autumn samples from each mulberry cultivar. (**A**) Venn diagrams illustrating the number of OTUs in spring and autumn. Abbreviations: CQ, CG, XL and HG represent fungal communities from “Chuan Sang No.7637”, “Changguo Sang”, “Xin Lunjiao”, and “Hong Guo No.2”, respectively. Different colors represent different groups. Values represent the number of OTUs. (**B**) Principal Coordinate Analysis (PCoA) based on Bray–Curtis dissimilarity metrics for all samples using ITS data. *n* = 3 for each sample. Spring samples and autumn samples are represented in green and yellow colors, respectively. Abbreviations: SH, SX, SQ, and SC represent fungal communities from “Hong Guo No.2”, “Xin Lunjiao”, “Chuan Sang No.7637”, and “Changguo Sang” in spring, respectively. AH, AX, AQ, and AC represent fungal communities from “Hong Guo No.2”, “Xin Lunjiao”, “Chuan Sang No.7637”, and “Changguo Sang” in autumn, respectively.

Relative to the spring samples, the number of specific OTUs in HG, XL, and CQ was notably lower in the autumn samples, decreasing from 340, 365, and 318 to 87, 152, and 105, respectively. However, in the cultivar CG, the number of unique OTUs in the spring samples (184) was almost equal to the number in the autumn (181), and the number of shared OTUs in both samples (222) was the highest among the four cultivars (Figure 6A). PCoA analysis also indicated that the samples for both seasons generally had a significant distance between each other. Interestingly, all spring samples were located in a similarity level along the first component (PCoA 1, 53.59%). Meanwhile, AQ, AX, and AH gathered closely based on the first component (PCoA 1, 53.59%) and the second component (PCoA 2, 14.79%). Whereas, AC was distinct from other autumn samples and close to SC, which had a higher PCoA 1 value (53.59%) and a lower PCoA 2 value (14.79%) (Figure 6B). Moreover, at the genus level, *Cladosporium* sp. accounted for 41.13, 51.71, 75.41 and 7.17% of the OTUs in AX, AH, AQ, and AC respectively. This was the most dominant OTU in the autumn samples for all cultivars with the exception of AC (data not given). Overall, seasonal shifts played a vital role in the composition of the mulberry fungal community, while those in CG exhibited relative stability (Figure 6B).

**Figure 7:**
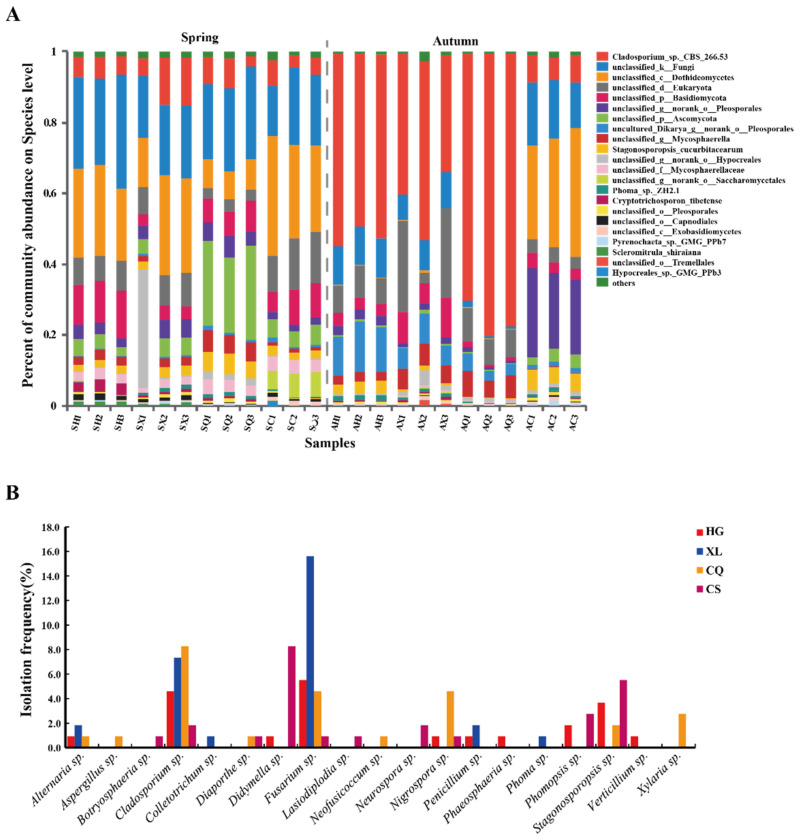
Taxonomic distribution and comparisons of diversity among the four mulberry cultivars (**A**) Distribution of taxa at the species-level based on ITS amplicons. *n* = 3 for each sample. Abbreviations: SH, SX, SQ, and SC represent fungal communities from “Hong Guo No.2”, “Xin Lunjiao”, “Chuan Sang No.7637”, and “Changguo Sang” in spring, respectively. AH, AX, AQ, and AC represent fungal communities from “Hong Guo No.2”, “Xin Lunjiao”, “Chuan Sang No.7637”, and “Changguo Sang” in autumn, respectively. (**B**) Distribution of cultured-isolate taxa at the genus level from four mulberry cultivars. The isolation frequency (IF) was calculated using the formula: IF (%) = the number of isolates of a certain taxa/the total number of isolates × 100%. Abbreviations: CQ, CG, XL, and HG represent fungal communities from “Chuan Sang No.7637”, “Changguo Sang”, “Xin Lunjiao”, and “Hong Guo No.2”, respectively. Different colors represent different groups.

Notably, based on the results from the culture-independent approach, *Scleromitrula shiraiana* was only identified in the susceptible mulberry cultivars, representing 1.32% and 0.07% in the SH and SX samples, respectively (Figure 7A), whereas no isolates of *Scleromitrula* sp. were found in the pure cultures collected by the culture method (Figure 7B), which may have been related to the low abundance of this genus. These data indicated that the causal pathogen of mulberry fruit sclerotiniosis was only detected in the endophytic fungal community of susceptible mulberry cultivars in the spring samples.

## 4. Discussion

Plants harbor diverse endophytes, many of which play a potential role in plant growth promotion and disease resistance [10,46]. It appears that both cultivars (genotypes) and environmental conditions significantly affect the composition of the endophytic fungi community of their plant hosts [44,47]. Determining the diversity of endophytic fungi in different cultivars may provide a basis for understanding their functional roles.

This study used both culture-dependent and culture-independent approaches to characterize the endophytic fungal community in four mulberry cultivars during spring and autumn. Differences in the fungal composition of the cultivars were identified between the two methods, especially at the genus level. A total of 19 genera were identified by culture methods and 40 by culture-independent NGS methods. Though sequences of 40 genera were obtained by a metagenomic approach, only 4 exhibited an average abundance over 1% and could be classified. Additionally, 3 of the 4 genera were also isolated by the culture-dependent method (Figure 7). While the NGS approach generated much greater diversity estimates, much of that diversity came from OTUs that had a low number of sequence reads in a few samples, and did represent major endophytic fungi [37]. Moreover, the functions of endophytic fungi, such as pathogenicity or mutualism, can rarely be predicted. Comparatively, the culture approach was not exhaustive, as just three media types were used and colonies were only selected based on morphology. For example, the isolation frequency of members of the *Hypocreales* (19.3%) was found to be the highest in autumn using the culture approach, but the relative abundance of its members was only 1.28% (Figure 4). A high isolation frequency using the culture method may only have indicated that they were easy to culture in the laboratory rather than reflect their abundance in hosts. It is evident that the culture-dependent method produces biased results for the richness and diversity of host microbiomes [48]. Therefore, a combination of NGS and culture-dependent methods can provide a powerful strategy for comprehensively investigating community diversity and the function of endophytic microbes.

Microbial communities in plants have demonstrated the ability to change across seasons, which was likely due to the seasonality of photosynthesis, precipitation, and temperature [28]. Walters et al. [49] reported that the relative abundance of members of the rhizosphere microbiome was associated with climate variations and that taxa within the *Pseudomonadaceae* responded negatively to long-term precipitation. Photosynthesis was reported to be one of the likely major drivers of shifts in the function of microbial communities in a coniferous forest and that the activity of ectomycorrhizal fungi was reduced in winter [50].

Seasonal shifts in climate also had a significant impact on community diversity, with the total number of OTUs decreasing from 813 in spring to 652 in autumn (Figure S3A). Considering taxonomic classification, the seasonality effect was much more pronounced in HG, XL, and CQ, where the diversity of the endophytic fungal community was higher in spring than in autumn (Figure 1). Notably, community diversity in CG, however, was not as highly affected by the change of seasons, with the total number of OTUs showing just a slightly decline from spring to autumn (*n* = 3) and the number of shared OTUs (*n* = 222) being the highest among all cultivars (Figure 6A). Pérez-Izquierdo et al. [51] found that the genotype of trees was crucial for shaping the structure of fungal communities in Mediterranean pine forests. Sun et al. [52] also reported that the host genotype contributed to 30.1% of the variability in the endophytic composition of three forest species (*Quercus liaotungensis, Ulmus macrocarpa,* and *Betula platyphylla*) in a mixed temperate forest. Liu et al. [53] also observed a distinct genotype influence on the composition of the endophytic fungal microbiota of apple cultivars and the relative abundance of specific genera or species. Similar results were found for *Alnus*, *Quercus ilex*, and *Picea mariana* [54,55,56]. The composition of the endophytic community in mulberry trees also varied according to host genotype as demonstrated in the present and previous studies [11].

Overall, host genotypes and environmental conditions interact to determine the assembly of beneficial or detrimental (pathogenic) members of the host microbiome [24]. Gehring et al. [57] demonstrated that tree genetics defines the fungal partner communities that potentially confer drought tolerance. Mendes et al. [26] determined that breeding for resistance may have unintentionally altered the rhizosphere microbiome composition, as well as the frequency of the occurrence of beneficial microorganisms. Interestingly, powdery mildew and wheat leaf rust infections are more severe in moist coastal areas of Australia compared to drier parts of the country [58]. In this study, the resistant cultivar CQ harbored more *Cladosporium* sp. (Figure 7B). Moreover, based on the results of the previous antifungal assay, we found that many isolates of this genus (e.g., AX1, AX15) exhibited varying degrees of antagonistic activity against *Sclerotinia sclerotiorum* PZ-2 (the main pathogen that causes mulberry fruit sclerotiniosis) (Data not given). It has been speculated that *Cladosporium* sp. contributes to the design and construction of beneficial microbial synthetic communities. Notably, the pathogen (*Scleromitrula shiraiana*) responsible for fruit sclerotiniosis that usually occurs in spring was only detected in the spring samples of susceptible mulberry cultivars (Figure 7A). The findings of this study might suggest that phytopathogens prefer to live in the susceptible varieties (HG, XL) rather than the resistant varieties (CQ and CG). The phenomenon showed that host genotypes (resistant or susceptible) can influence the presence or colonization of beneficial fungi or phytopathogens, suggesting that there is a certain relationship between the incidence of mulberry fruit sclerotiniosis and the endophytic fungal microbiome. In addition to mulberry, the plant–microbe interaction and the role of microbes in plant stress tolerance were also reported on beans [26] and other plants [59,60,61]. Interestingly, no significant changes in the composition of the fungal community were observed in the resistant cultivar CG, suggesting that it may possess a stable and beneficial microbiome with members that have environmental tolerance and antagonistic activity that may help prevent fruit sclerotiniosis. This premise should be explored further.

## 5. Conclusions

A combination of high-throughput sequencing (NGS) and culture-dependent methods were used to investigate the endophytic fungal community during the spring and autumn of four mulberry cultivars with different levels of resistance to mulberry fruit sclerotiniosis. The results indicated that significant variations in community composition and diversity occurred in different mulberry cultivars mainly due to seasonal changes, followed by host genotypes. Moreover, the resistance of mulberry cultivars might affect the colonization of the pathogen to certain extent. This study broadens our understanding of the factors modulating the microbiome of mulberry trees and provides information that could see mulberry endophytes used as biological control agents to facilitate host resistance and growth. It will be important, however, to determine the manner in which the microbial assemblages associated with the different mulberry cultivars are established and their stability, before they can be associated with, and potentially exploited for, improving stress tolerance and disease resistance.

## Data Availability

Complete culture-independent sequence data sets were submitted to the NCBI Short Read Archive (SRA) database under the accession number SRP165744. This data can be found at https://www.ncbi.nlm.nih.gov/sra/SRX4886026[accn]. The ITS gene sequences of the cultured fungal isolates were submitted to GenBank under the accession numbers MH884069-MH884177. All data generated or analyzed during this study are included in this article and its supporting information files.

## Supplementary Materials

The following are available online at https://www.mdpi.com/article/10.3390/microorganisms9102052/s1. Table S1: Alpha diversity analysis of the endophytic fungal communities of four different mulberry cultivars in spring and autumn. Table S2: Cumulative list of cultivable endophytic fungi in mulberry and their taxonomic information. Figure S1: Rarefaction curves depicting the number of OTUs identified in each sample using a 97% similarity. The *x*-axis indicates the number of sequences obtained from each sample. The *y*-axis represents the number of OTUs observed based on the Sobs index. *n* = 3 for each sample. Different colors represent different groups. Abbreviations: SH, SX, SQ, and SC represent fungal communities from “Hong Guo No.2”, “Xin Lunjiao”, “Chuan Sang No.7637”, and “Changguo Sang” in spring, respectively. AH, AX, AQ, and AC represent fungal communities from “Hong Guo No.2”, “Xin Lunjiao”, “Chuan Sang No.7637”, and “Changguo Sang” in autumn, respectively. Figure S2: Hierarchical clustering of the endophytic fungal communities in different seasons and in different mulberry cultivars. The length of the branches represents the distance between the samples. *n* = 3 for each sample. Different colors represent different groups. Abbreviations: SH, SX, SQ, and SC represent fungal communities from “Hong Guo No.2”, “Xin Lunjiao”, “Chuan Sang No.7637”, and “Changguo Sang” in spring, respectively. AH, AX, AQ, and AC represent fungal communities from “Hong Guo No.2”, “Xin Lunjiao”, “Chuan Sang No.7637”, and “Changguo Sang” in autumn, respectively. Figure S3: Venn diagram illustrating the number of OTUs obtained in different seasons and in different cultivars. (**A**) Grouped by seasons (spring and autumn). (**B**) Grouped by cultivars. CQ, CG, XL, and HG represent fungal communities from “Chuan Sang No.7637”, “Changguo Sang”, “Xin Lunjiao”, and “Hong Guo No.2”, respectively. Different colors represent different groups. Values represent the number of OTUs.
