# Supplementary material for: Seasonal Characterization of the Endophytic Fungal Microbiome of Mulberry (Morus spp.) Cultivars Resistant and Susceptible to Sclerotiniosis
Source: Microorganisms. 2021 Sep 28;9(10):2052. doi: 10.3390/microorganisms9102052 (PMC8537754; doi:10.3390/microorganisms9102052)
Supplement: Supplementary file 1 [file microorganisms-09-02052-s001.zip › microorganisms-1371267-supplementary.pdf]

Table S1. Alpha diversity analysis of the endophytic fungal communities of four different mulberry cultivars in spring and autumn.

Samples	Sobs index	Chao index	Shannon index	Simpson index
Spring samples				
SH	353.33 ± 9.45 b	414.15 ± 32.54 b	4.06 ± 0.13 a	0.04 ± 0.00 d
SX	392.00 ± 15.10 a	499.73 ± 54.09 a	3.93 ± 0.32 a	0.06 ± 0.05 d
SQ	321.00 ± 9.54 b	400.69 ± 63.51 b	3.74 ± 0.07 a	0.05 ± 0.00 d
SC	264.67 ± 51.64 c	307.17 ± 82.84 c	3.70 ± 0.10 a	0.05 ± 0.00 d
Autumn samples				
AH	171.33 ± 4.93 e	206.40 ± 19.54 cd	2.20 ± 0.05 d	0.29 ± 0.02 b
AX	210.33 ± 23.76 d	241.71 ± 16.96 cd	2.60 ± 0.19 c	0.20 ± 0.06 c
AQ	135.67 ± 3.79 e	151.42 ± 2.70 d	1.21 ± 0.21 e	0.58 ± 0.07 a
AC	255.00 ± 5.57 c	303.44 ± 34.72 c	3.26 ± 0.18 b	0.10 ± 0.01 d

Abbreviations: SH, SX, SQ, and SC represent fungal communities from 'Hong Guo No.2', 'Xin Lunjiao', 'Chuan Sang No.7637', and 'Changguo Sang' in spring, respectively. AH, AX, AQ, and AC represent fungal communities from 'Hong Guo No.2', 'Xin Lunjiao', 'Chuan Sang No.7637', and 'Changguo Sang' in autumn, respectively. Data represent the mean ± standard deviation (*SD*) of three independent replicates. Means followed by different letters are significantly different at $P < 0.05$.

Table S2. Cumulative list of cultivable endophytic fungi in mulberry and their taxonomic information.

classification		cultivars								total
level	taxa	SH	SX	SQ	SC	AH	AX	AQ	AC	
phyla	*Ascomycota*	9	13	12	7	14	18	16	20	109
class	*Dothideomycetes*	6	4	7	1	6	7	6	18	55
	Sordariomycetes	2	9	4	6	8	9	10	2	50
	Eurotiomycetes	1	0	1	0	0	2	0	0	4
order	*Botryosphaeriales*	0	0	1	0	0	0	0	2	3
	Capnodiales	1	2	3	1	4	6	6	1	24
	Diaporthales	2	0	1	4	0	0	0	0	7
	Eurotiales	1	0	1	0	0	2	0	0	4
	Glomerellales	0	0	0	0	0	1	0	0	1
	Hypocreales	0	9	0	0	7	8	5	1	30
	Pleosporales	5	2	3	0	2	1	0	15	28
	Sordariales	0	0	0	2	0	0	0	0	2
	Trichosphaeriales	0	0	0	0	1	0	5	1	7
	Xylariales	0	0	3	0	0	0	0	0	3
family	*Aspergillaceae*	1	0	1	0	0	2	0	0	4
	Botryosphaeriaceae	0	0	1	0	0	0	0	2	3
	Cladosporiaceae	1	2	3	1	4	6	6	1	24
	Diaporthaceae	0	0	1	1	0	0	0	0	2
	Didymellaceae	3	1	2	0	2	0	0	15	23
	Glomerellaceae	0	0	0	0	0	1	0	0	1
	Nectriaceae	0	9	0	0	6	8	5	1	29
	Phaeosphaeriaceae	1	0	0	0	0	0	0	0	1
	Pleosporaceae	1	1	1	0	0	1	0	0	4
	Sordariaceae	0	0	0	2	0	0	0	0	2
	Trichosphaeriaceae	0	0	0	0	1	0	5	1	7
	Valsaceae	2	0	0	3	0	0	0	0	5
	Xylariaceae	0	0	3	0	0	0	0	0	3
	incertae sedis	0	0	0	0	1	0	0	0	1
genus	*Alternaria*	1	1	1	0	0	1	0	0	4
	Aspergillus	0	0	1	0	0	0	0	0	1
	Botryosphaeria	0	0	0	0	0	0	0	1	1
	Cladosporium	1	2	3	1	4	6	6	1	24
	Colletotrichum	0	0	0	0	0	1	0	0	1
	Diaporthe	0	0	1	1	0	0	0	0	2
	Didymella	1	0	0	0	0	0	0	9	10
	Fusarium	0	9	0	0	6	8	5	1	29
	Lasiodiplodia	0	0	0	0	0	0	0	1	1

Neofusicoccum	0	0	1	0	0	0	0	0	1
Neurospora	0	0	0	2	0	0	0	0	2
Nigrospora	0	0	0	0	1	0	5	1	7
Penicillium	1	0	0	0	0	2	0	0	3
Phaeosphaeria	1	0	0	0	0	0	0	0	1
Phoma	0	1	0	0	0	0	0	0	1
Phomopsis	2	0	0	3	0	0	0	0	5
Stagonosporopsis	2	0	2	0	2	0	0	6	12
Verticillium	0	0	0	0	1	0	0	0	1
Xylaria	0	0	3	0	0	0	0	0	3

Abbreviations: SH, SX, SQ, and SC represent fungal communities from 'Hong Guo No.2', 'Xin Lunjiao', 'Chuan Sang No.7637', and 'Changguo Sang' in spring, respectively. AH, AX, AQ, and AC represent fungal communities from 'Hong Guo No.2', 'Xin Lunjiao', 'Chuan Sang No.7637', and 'Changguo Sang' in autumn, respectively.

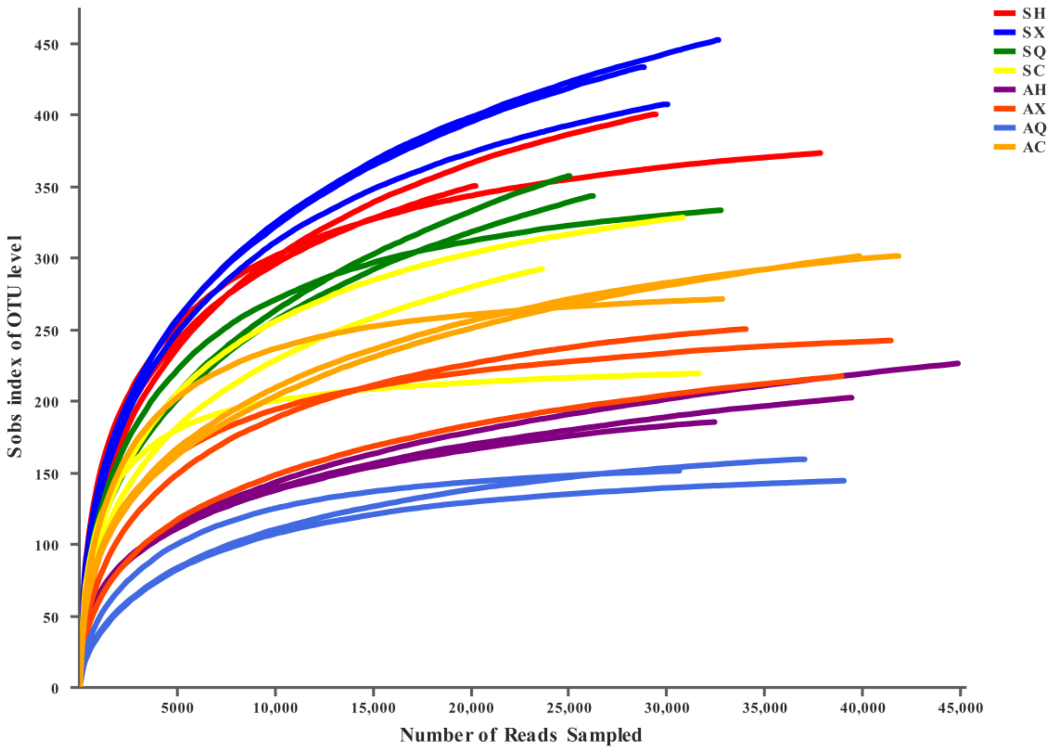

Figure S1. Rarefaction curves depicting the number of OTUs identified in each sample using a 97% similarity. The x-axis indicates the number of sequences obtained from each sample. The y-axis represents the number of OTUs observed based on the Sobs index. N=3 for each sample. Different colors represent different groups. Abbreviations: SH, SX, SQ, and SC represent fungal communities from 'Hong Guo No.2', 'Xin Lunjiao', 'Chuan Sang No.7637', and 'Changguo Sang' in spring, respectively. AH, AX, AQ, and AC represent fungal communities from 'Hong Guo No.2', 'Xin Lunjiao', 'Chuan Sang No.7637', and 'Changguo Sang' in autumn, respectively.

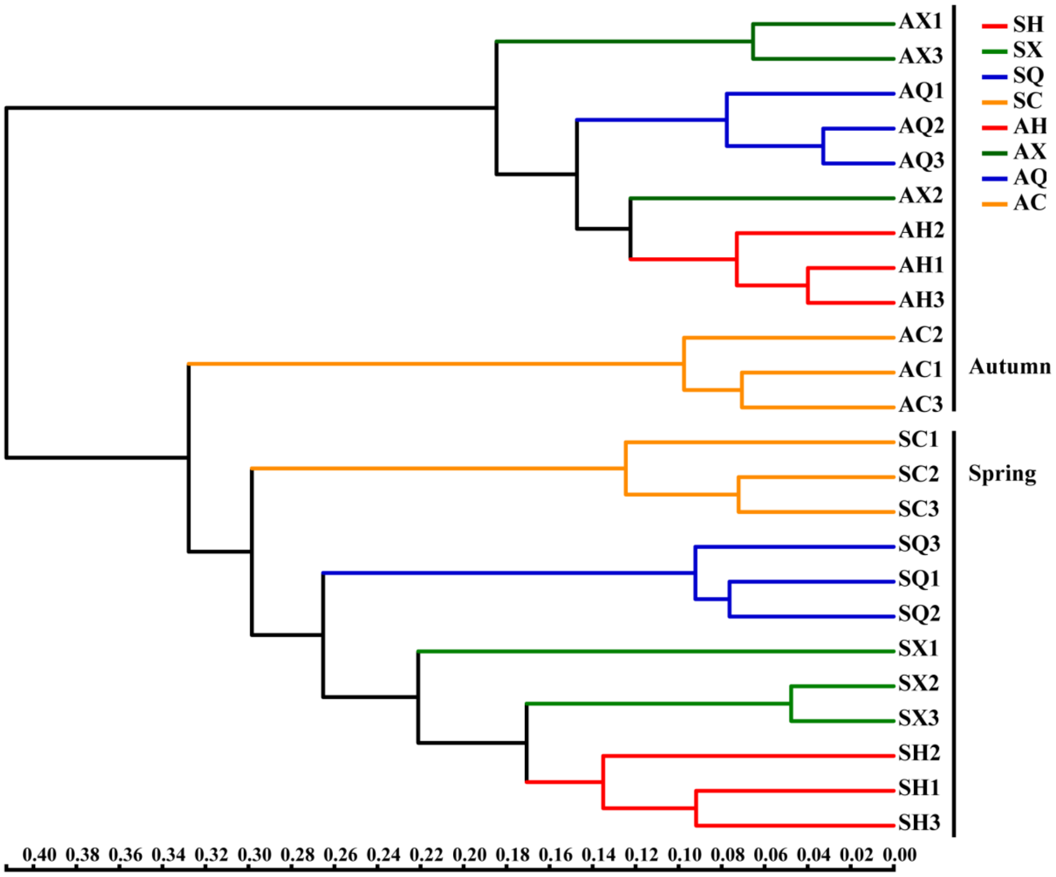

Figure S2. Hierarchical clustering of the endophytic fungal communities in different seasons and in different mulberry cultivars. The length of the branches represents the distance between the samples. N=3 for each sample. Different colors represent different groups. Abbreviations: SH, SX, SQ, and SC represent fungal communities from 'Hong Guo No.2', 'Xin Lunjiao', 'Chuan Sang No.7637', and 'Changguo Sang' in spring, respectively. AH, AX, AQ, and AC represent fungal communities from 'Hong Guo No.2', 'Xin Lunjiao', 'Chuan Sang No.7637', and 'Changguo Sang' in autumn, respectively.

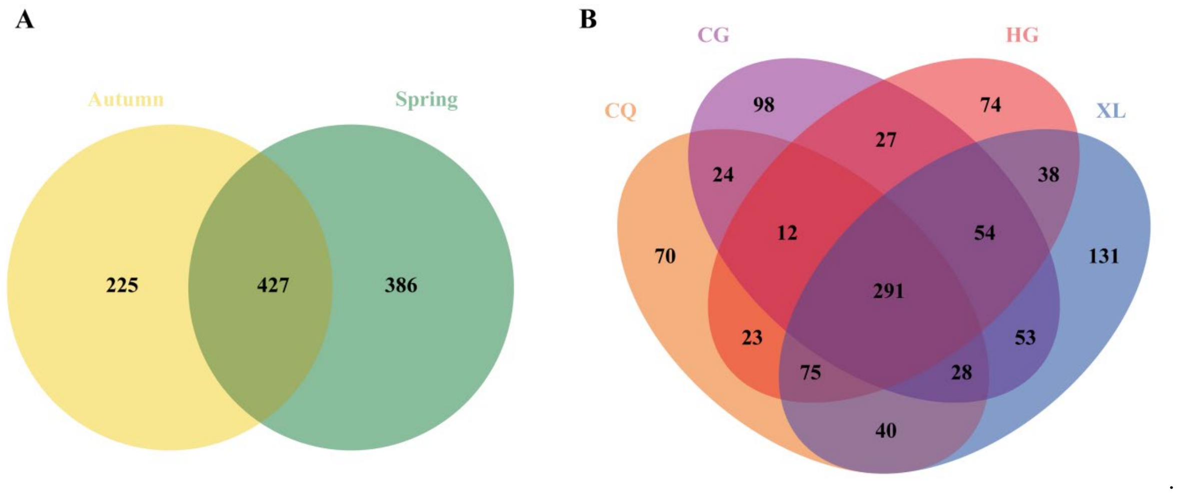

Figure S3. Venn diagram illustrating the number of OTUs obtained in different seasons and in different cultivars. (A) Grouped by seasons (spring and autumn). (B) Grouped by cultivars. CQ, CG, XL, and HG represent fungal communities from 'Chuan Sang No.7637', 'Changguo Sang', 'Xin Lunjiao', and 'Hong Guo No.2', respectively. Different colors represent different groups. Values represent the number of OTUs.
